# Convolutional Networks Outperform Linear Decoders in Predicting EMG From Spinal Cord Signals

**DOI:** 10.3389/fnins.2018.00689

**Published:** 2018-10-17

**Authors:** Yi Guo, Sinan Gok, Mesut Sahin

**Affiliations:** ^1^Independent Researcher, Venice, CA, United States; ^2^Neural Prosthetics Laboratory, Department of Biomedical Engineering, New Jersey Institute of Technology, Newark, NJ, United States

**Keywords:** machine learning, artificial neural network, convolutional neural network, corticospinal tract, microelectrode array, signal processing, neural signal decoding

## Abstract

Advanced algorithms are required to reveal the complex relations between neural and behavioral data. In this study, forelimb electromyography (EMG) signals were reconstructed from multi-unit neural signals recorded with multiple electrode arrays (MEAs) from the corticospinal tract (CST) in rats. A six-layer convolutional neural network (CNN) was compared with linear decoders for predicting the EMG signal. The network contained three session-dependent Rectified Linear Unit (ReLU) feature layers and three Gamma function layers were shared between sessions. Coefficient of determination (*R*^2^) values over 0.2 and correlations over 0.5 were achieved for reconstruction within individual sessions in multiple animals, even though the forelimb position was unconstrained for most of the behavior duration. The CNN performed visibily better than the linear decoders and model responses outlasted the activation duration of the rat neuromuscular system. These findings suggest that the CNN model implicitly predicted short-term dynamics of skilled forelimb movements from neural signals. These results are encouraging that similar problems in neural signal processing may be solved using variants of CNNs defined with simple analytical functions. Low powered firmware can be developed to house these CNN solutions in real-time applications.

## 1. Background and significance

Multi-electrode arrays (MEAs) are pushing the limits of technology to increase the number of channels and thereby expand the size of neural data collected in a single experimental session (Viventi et al., 2011; Xu et al., 2017). As the amount of data increases, manual review and analysis of neural data will become increasingly difficult. New electrodes and hardware will make it a pressing necessity for automated processing of large quantity of neural data. As a result, attention has been turning to machine learning for answers. Recently Deep Learning (DL) has emerged as a particularly promising paradigm.

DL is a machine learning technique using Artificial Neural networks (ANNs) with multiple hidden layers. In the past decade, ANNs has made significant breakthroughs in image recognition (He et al., 2015; Rastegari et al., 2016) and natural language processing (Amodei et al., 2015; Lipton, 2015; Wu et al., 2016). Due to their origins as connectionist models of neural circuits (Hopfield, 1982; Hopfield and Tank, 1985), ANN can naturally simulate the types of processing performed by the nervous system. In addition, ANNs can be easily parallelized as demonstrated by various large-scale applications (Dean et al., 2012; Abadi et al., 2016). These traits will make ANN based approaches increasingly relevant to neuroscience.

ANN model parameters are estimated by computing gradients: numerical partial derivative of output error (costs) relative to model parameters. The process is modular: Each layer of the ANN only needs to define the derivative of its output relative to its inputs and parameters within the layer, and the partial derivative of any parameter in the model can be computed via the Chain Rule in a process known as back propagation (Hecht-Nielsen, 1989; Chauvin and Rumelhart, 1995). Each layer of an ANN contains an activation function, allowing growing stacks of layers to approximate increasingly complex non-linear functions. ANNs for commercial applications like image classifiers can have hundreds of layers (Bengio, 2013; LeCun et al., 2015), allowing them to represent non-linear transformations between sets of data.

DL is starting to see adoption in many areas of neural signal processing such as in EEG classification tasks (Schirrmeister et al., 2017), Epilepsy Detection (Gadhoumi et al., 2016), Visual Presentation based BCI (Shamwell et al., 2016), and cognitive performance measurement (Hajinoroozi et al., 2016). The approach as been particularly successful in decoding motor activity and imagery with discrete classes from EEG signals (Ren and Wu, 2014; Sakhavi et al., 2015; Stober, 2017; Tabar and Halici, 2017; Tang et al., 2017; Lawhern et al., 2018). There were notably four brain-computer-interface (BCI) competitions hosted by BBCI group of Berlin Institute of Technology since 2003 (Sajda et al., 2003; Blankertz et al., 2004; Müller et al., 2004). In 2016, a group at Stanford and Google developed Latent Factor Analysis via Dynamic Systems (LFADS) (Sussillo et al., 2016), an auto-encoder that uses recurrent networks for both encoding and decoding stages in order to extract underlying dynamics from spike trains, representing a major step forward in applying ANNs to problems in neuroscience. Applications of DL in neuroscience will gain popularity as other investigators are encouraged by these initial success.

This paper demonstrates a method of solving the decoding problem where the output is a continuous valued signal (EMG bursts) using unsorted Multi-Unit Activity (MUA) as inputs. It is a difficult problem analytically because one cannot picture how neural signals might explain the EMG activity by visual inspection **Figure 2**. This is supported by the relatively poor performance of linear decoders.

Convolutional ANNs are of particular interest because they allow the relations between signals to be described as time-invariant filters. Each layer draws non-linear features from outputs of the previous layer. Because inputs are padded only from the front **Figure 3**, the ANN captures consistent causal relations between inputs (neural signal envelope) and output (EMG envelope). Larger network depths increase the types of non-linearities that can be represented. Reconstruction of EMG bursts could be achieved by a convolutional network comprised of six layers. This depth was empirically determined to be appropriate for the complexity of the available data, performance of network over six layers quickly plateaued due to diminishing returns. (see **Figure 10** and discussions).

The spinal cord was chosen as a testbed because it is not known to contain any memory function and therefore should have short impulse response as a system. Many regions of the brain, in contrast, contain recurrent connections allowing them to persist in a memory state until perturbed. Identifying such systems will require autoregressive or recurrent models.

The datasets used were recorded as a part of a proof-of-concept study for Spinal Cord-Computer Interfaces (SCCI) (Gok and Sahin, 2016, 2017). The SCCI as a neural interface was proposed by our group (Prasad and Sahin, 2006, 2010), which extracts volitional motor information from descending tracts of the spinal cord above the level of injury site. The SCCI approach is motivated by a number of advantages compared to traditional cerebral interfaces. First and foremost, SCCI can potentially tap into command signals at the spinal cord level where descending signals from many cerebral cortices converge.

## 2. Materials and methods

### Data collection

**Figure 1:**
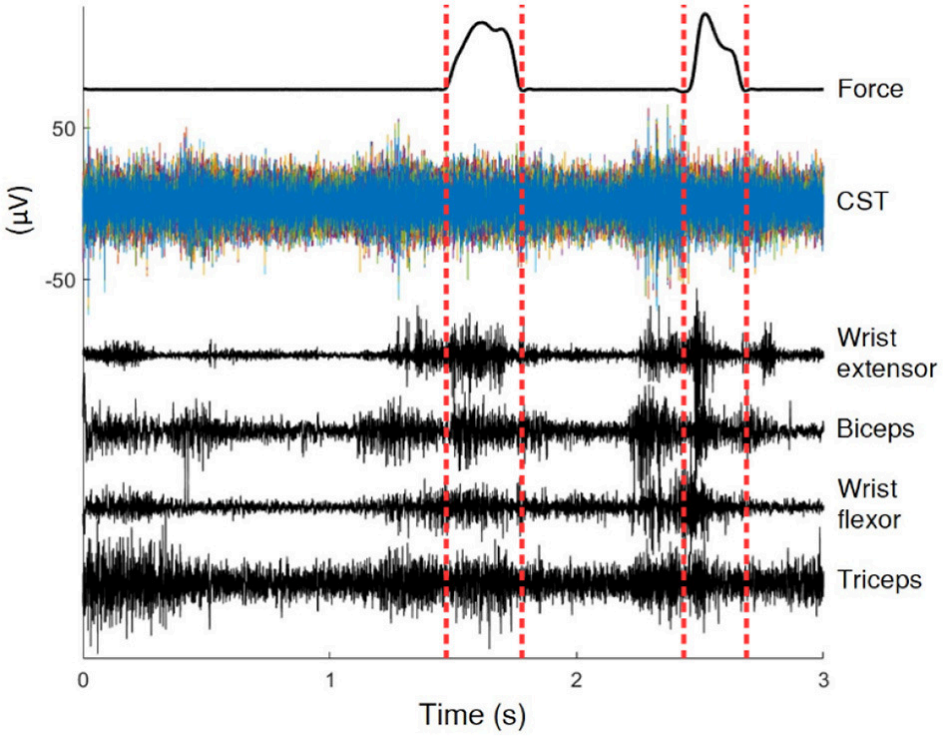
Recordings of a typical trial. In this 3 s trial the animal was in contact with the rod for less than 0.5 s, the interval marked by dashed lines. The position is only known within the interval.

Rats were trained for a reach-to-pull task inside a Plexiglas box with a 3x1cm window on the wall. A vertical steel rod attached to a force transducer was located just outside the window. Animals reached with one forelimb through the window and pulled on the rod with sufficient force to be rewarded with sugar pellets. Refer to Gok and Sahin (2016) for details of experimental apparatus. Three to four seconds during each pull were recorded as a trial, centered about the time of contact of the hand with the rod. The animal's paw was typically in contact with the rod for less than a second per trial (averaged approx. 500 ms) (Dashed Vertical Lines in Figure 1), and the remainder of the data represented unconstrained movements to and away from the rod. Animals performed over hundred successful trials per day. The current analysis used all the data, over 80% of which were unrestricted. Figure 1 shows raw signals from a typical trial.

To collect command signals from the spinal cord, after dorsal laminectomy, a 4 × 7 planar MEA was inserted vertically into the dorsal median sulcus between the two halves of the spinal cord, with the MEA contacts facing the direction of the trained hand at C4 level. As a result the electrode contacts were made at the ventral most part of the dorsal column (Guo et al., 2014; Gok and Sahin, 2016, 2017). The ribbon cable of the MEA was attached to an Omnetics micro connector held above the spinal cord by a stainless steel wireframe anchored to the spinous processes on the C2 and C5 vertebra. Teflon coated 25um stainless steel wires collected EMG from the bicep and triceps muscles, as well as a wrist flexor and an extensor muscle. These wires were subcutaneously tunneled through a metal tube to the top of the head to a second Omnetics micro connector fixed to the skull using dental acrylic and metal screws. For rat 1 and 2, both neural and EMG signals were acquired at 16 kHz using Triangular Biosystems (TBSI) wireless head stages. For rat 3 and 4, the signals were collected at 30 kHz using the Ripple Inc.'s (Salt Lake City, UT) tethered amplifier. Data collected from the two later animals had less crosstalk and were of a higher quality.

### Pre-processing

**Figure 2:**
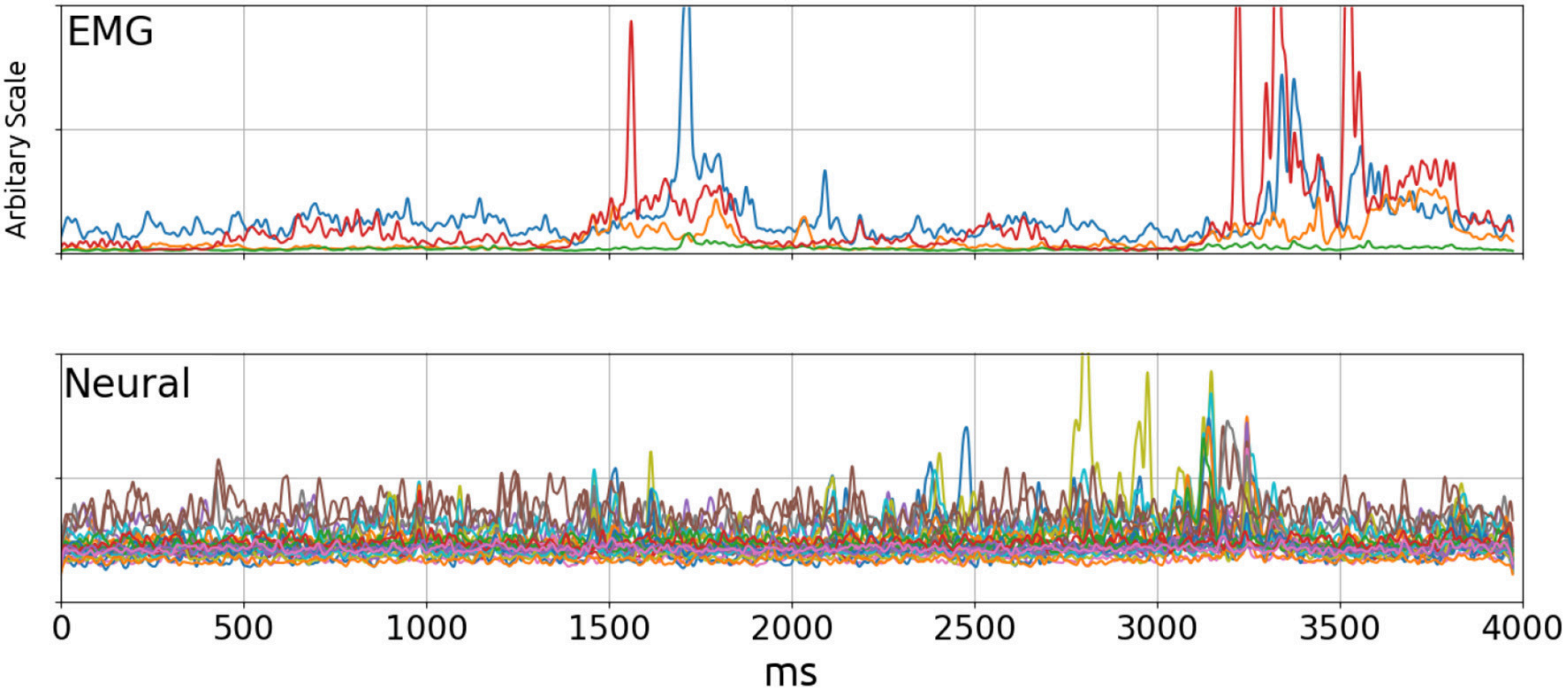
EMG **(Top)** and Neural **(Bottom)** signal envelopes from a trial. By visual inspection, there did not appear to be a significant correlation between the two signals. The poor performance of linear decoders testifies to the lack of correlation. Top: Blue, Wrist flexor; Orange, Biceps; Green, Triceps; Red, Wrist Extensor.

Neural signals from rat 1 and 2 were band-pass filtered at 20 Hz–3,500 Hz by the built-in filter in the amplifiers before digitization and another 20 Hz 3rd order zero-phase high-pass Butterworth filter before finding the envelopes to remove any remaining movement artifacts. Envelopes for both neural and EMG signals were computed by taking the square root of the square of the signal filtered with a Gaussian kernel (σ = 10*ms*). In the frequency domain, this filter is another Gaussian centered on zero with σ = 100/π*Hz* and a half-power frequency of approximately 37.5 Hz. Therefore the estimated evelopes are the RMS power of the signal, and the Gaussian kernel provided the smoothing function. Figure 2 shows a typical trial at this stage. The EMG Envelopes were further low-pass filtered using a third order zero-phase low-pass filter at 5–1 Hz to make it less uneven (5Hz: **Figures 6**, **7**; 1Hz: **Figure 9**; 5–1 Hz: **Figure 8**). Filtered signal envelopes were then down-sampled to 100 Hz. The final pre-processed inputs were 4-s trials containing 27 neural and 4 EMG channels collected simultaneously.

A session contains varying number of trials collected on the same day. Trials were divided into sequential groups of five. The first 3 trials from each group were assigned to the Training Set, and the next 2 were assigned to Cross-Validation Sets A and B, and the process was repeated for the remaining trials. This resulted in 60, 20, and 20% trials assigned to Training, Test, and Validation Sets, respectively. See Cross-Validation for Details.

Training was repeated 16 times in each session and the highest values were reported (see Cross Validation for details and justification), During the repeated training there was no randomization in trial assignments. At each repetition the same trials were assigned to Training and Cross-Validation Sets. The only difference between training attempts were the initial condition of the network.

### Network configuration

**Figure 3:**
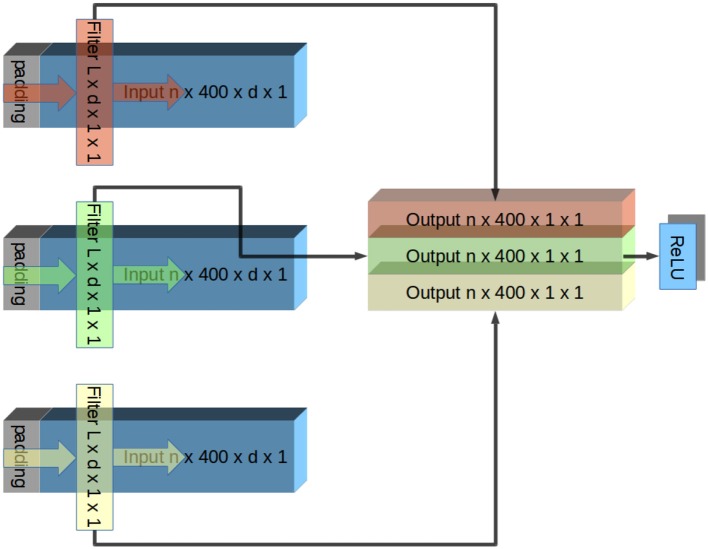
Illustration of one layer of CNN. The input signal has n trials, each containing d channels. In this illustration, this input is padded and convolved with a different filter per output channel. Each filter has an identical width as inputs (d), and therefore the convolution produces a single channel of output. Because the filters apply identically to each trial, the output channel has the same number of trials as the input. Output channels are grouped together and fed into element-wise ReLU rectification. Output of ReLU is the input to the next layer. This repeats until the output is a single channel, which is compared to a low-pass filtered EMG signal. Convolution in Tensorflow proceeds right to left due to conventions of CNNs. To account for this, inputs are flipped before the convolution and outputs are flipped again afterwards.

The network layers designs were such that they were convolutional in the time domain and densely connected in the spatial domain. This design was based on the assumption that there are little hierarchical spatial structure in the data because the contacts were located in a plane parallel to the fibers. This rationale is similar to one presented in Schirrmeister's paper (Schirrmeister et al., 2017). Each layer contained one convolutional filter per output channel. Each filter had the same width as the number of input channels and a length specified by its network and layer number. Inputs were padded in front to the same length to ensure that each layer was causal, and the output channels had the same lengths as the input channels (# of trials in session × 400 time bins × 1 output width × 1 channel). These outputs grouped together to form inputs to element-wise leaky ReLU (Gu et al., 2015) activation function (Rectified Linear Units) to extract features. Outputs of the ReLU served as inputs to the next layer. This organization is shown in Figure 3.

Networks were comprised of six layers. The first three were session-dependent feature layers. They had a length of 1 in the time dimension (10 ms) and the widths of 27 → 21 → 15 → 9 (denoting that the first layer had 27 inputs and 21 outputs, and so on). As a result all sessions from the same animal were transformed into 9-dimentional trials and concatenated to form a single data set. These layers were intended to compensate for day-to-day electrode micromotions by transforming neural envelopes into an identical set of 9 features that were assumed to exist in all sessions. This design was based on the prediction that the actual dimensionality of motor command signals was much lower than the number of recorded signals (Narayanan, 2005). The redundancy is also evident in the relatively small number of signals required for a successful BCI.

Two configurations for CNN were examined in this study to test the effectiveness of Convolutional Kernels with a closed form. Reconstructions were produced from the concatenated feature vectors computed in the previous step using 3 additional session-invariant layers. In both configurations they had the widths of 9 → 6 → 3 → 1. In the naive configuration, there were no assumptions on the shape of the filters. Each layer had the length of 10 time bins (100 ms) and there were no restrictions on their values. An alternative solution was devised to reduce the number of parameters to be estimated by Feature Engineering—specifically, describing the connection between layers using Gamma functions. Details of the implementation are explained in the following subsection.

The number of layers in the network was determined empirically. The performance improved with an added second and third layer. However it plateaued quickly due to diminishing returns **Figure 10**. The performance with twice as many layers were not noticeably different. In addition, very deep networks with over 10 layers were vulnerable to being stuck due to vanishing gradient. Batch Normalization and Residual Connections allowed training of these networks but there were no benefit to the additional complexity.

### Feature engineering

In a Gamma function layer the connection between each pair of input and output channels is defined by the closed form in 6. It is formulated to allow back-propagation of errors and prevent covariance drifts from layer to layer. The gamma function filter has a length of 300 ms (30 time bins), with the initial time to peak and delay randomized in the order of 10 ms. The function is completely specified by three parameters: Area under the curve (α), time to first non-zero point (*t*_0_) and inverse of the time constant (β). β and *t*_0_ were defined as exponents of TensorFlow variables so they are always positive. θ is a constant determining the sharpness of the corner and was set to 10 in this study.

(1) $$\begin{array}{l} t_{f}=\frac{1}{\theta \beta }\ln\left[\exp\left[\theta \beta \left(t-t_{0}\right)\right]+1\right] \end{array}$$

(2) $$\begin{array}{l} F\left(t\right)=\alpha \beta^{2}t_{f}\exp\left(-\beta t_{f}\right) \end{array}$$

Gamma functions are defined as functions of the transformed time variable *t*_*f*_, where *t*_*f*_ is a SoftPlus function of *t* and *t*_0_, a smoothed version of the ReLU. SoftPlus was used instead of ReLU because gradient of ReLU at 0 is technically complicated. Using SoftPlus, *t*_*f*_ is differentiable with respect to the time delay variable *t*_0_ for every point *t* on the filter. Unlike ReLU, output of a SoftPlus function is strictly greater than zero. This meant adding an ϵ is not necessary to prevent division by zero errors caused by infinite gradients. The Exponential Linear Unit (ELU) provides a possible alternative but it was not tested in this project.

**Figure 4:**
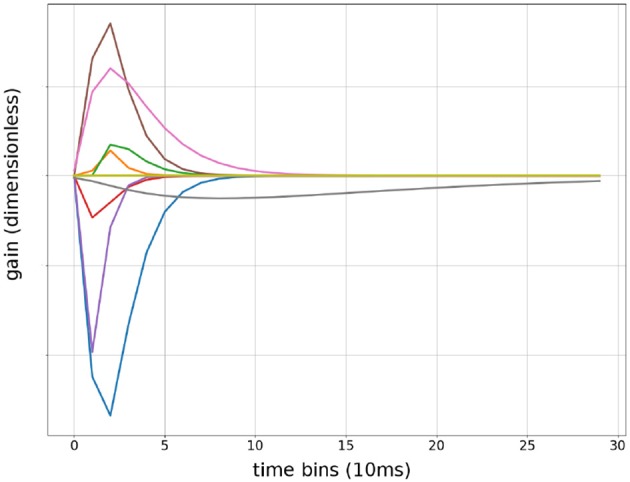
Sample of convolutional Kernels: debug output of input weights to a randomly chosen first layer units from 9 input features. Time is in bins of 10 ms, up to 300 ms.

The Gamma function has two terms: *t*_*f*_ multiplied with exp (−*βt*_*f*_) both of which are differentiable with respect to *t*_*f*_. Therefore, automatic gradient can be computed from *F*(*t*) back to α,β and *t*_0_ for all *t*. The factor β^2^ maintained constant area under the curve when β changed. This allowed the optimizer to independently adjust the length of the Gamma function without changing variance of the output. Because the area under the curve is always α, only α needed Xavier Initialization to prevent covariance drifts. β and *t*_0_ can be initialized randomly within their range. Convolutional Kernels were plotted after training to confirm the network had learned Gamma functions Figure 4. β values does not reflect sensitivity to particular frequencies because the convolution operates on evenlopes rather than the raw signal.

### Training

For a given EMG signal, the optimization error was defined as the sum of squares of the difference of reconstructed and low-pass-filtered EMG across all time points excluding the first second for all trials in a session. It was removed to prevent solutions using the transient response of the network. Each EMG signal was treated independently. Coefficient of correlation and coefficient of determination were simultaneously computed for the training set and the Cross Validation Sets but these values did not participate in the optimization.

The network was implemented using Tensorflow 1.2 and python 3.3. GPU processing was performed using a single GTX 1080 using CuDA 8.0 and CuDNN 6. Optimizations were performed using an adaptive moment estimation (ADAM) optimizer with a learning rate of 0.1. The remaining optimizer parameter retained their default values in Tensorflow (*B*_1_ = 0.9, *B*_2_ = 0.999, ϵ = 10^−8^). The number of training steps were 1,000. This number of steps was sufficient to over-fit in vast majority of training sequences: *R*^2^ values were not the highest at the last step for non-training group A and B. L1 and L2 regularizations were disabled because using an early stop (choosing a model from a step before 1,000) with a validation set was more effective.

### Cross validation

Training is an optimization process; the result can vary significantly depending on initial condition. To account for this, 16 attempts were made for each EMG signal in every session. To determine the point of over-fit, cross-validation was used. During a training attempt, every step can be considered a different model. Only the models where *R*^2^ were the highest for group A and B were retained as model *A*_max_ and *B*_max_, respectively. Neither Set A nor B were included in training, so they can both be considered Validation Sets and be used to determine the optimal step to end the training.

**Figure 5:**
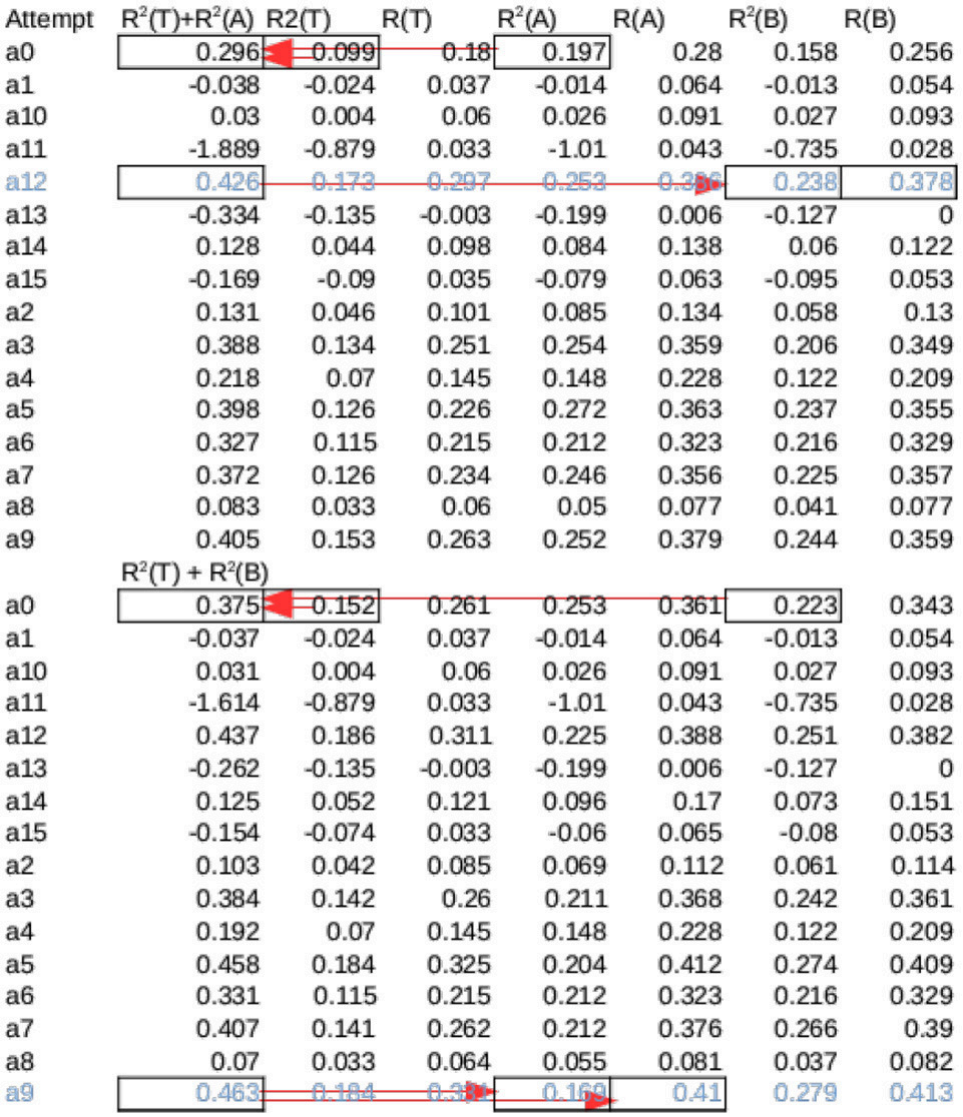
Cross validation for a session from Rat 15—The same session in Figure 7, marked with black arrow in Figure 6B. Training was attempted 16 times from different initial conditions (a0–a15). Each attempt produced 2 Models, *A*_max_ and *B*_max_, which had the maximum overall *R*^2^ across all sessions for set A and B, respectively. The sum of *R*^2^ of each *A*_max_ for set A and Test were summed (marked by arrows on a0), and the model with the highest sum was tested on B (marked by arrows on a13). Likewise, the model among *B*_max_ with the highest sum of *R*^2^ for set B and Test was tested on A. The average of these two values were the overall performance for the session. Note that this is not necessarily the highest possible *R*^2^, as seen in the first half of the table.

Because Set A and B have no overlap, they can also both be considered as the test set when the other set is used as Validation set to choose a stopping point. To compute performances using B as the test set, one model in each session was chosen among the 16 *A*_max_ models to be tested on Set B without using any information from Set B. *A*_max_ Models were sorted by the sum of the *R*^2^ values for Training Set and Set A and the model with the highest sum was chosen. Only this model was tested on group B, and its performance was defined as *A*_max_ (*B*) for that session. Likewise a single *B*_max_ model was chosen for the sum of its *R*^2^ values on Training and Set B, and its performance was defined as *B*_max_ (*A*). To account for difference in data qualities, the overall performance of a session is the mean of *A*_max_ (*B*) and *B*_max_ (*A*) for that session. Figure 5 illustrates this selection process. The 16 attempts should not be considered sample drawn from a random distribution because only one model was selected for testing without knowledge of the test set. Because there were no pretraining, an attempt either converges or not, this can be seen in column 5 and 7 in Figure 5. as a result the mean *R*^2^ value over all attempts is usually close to 0. For this reason the mean performances are not meaningful.

**Figure 6:**
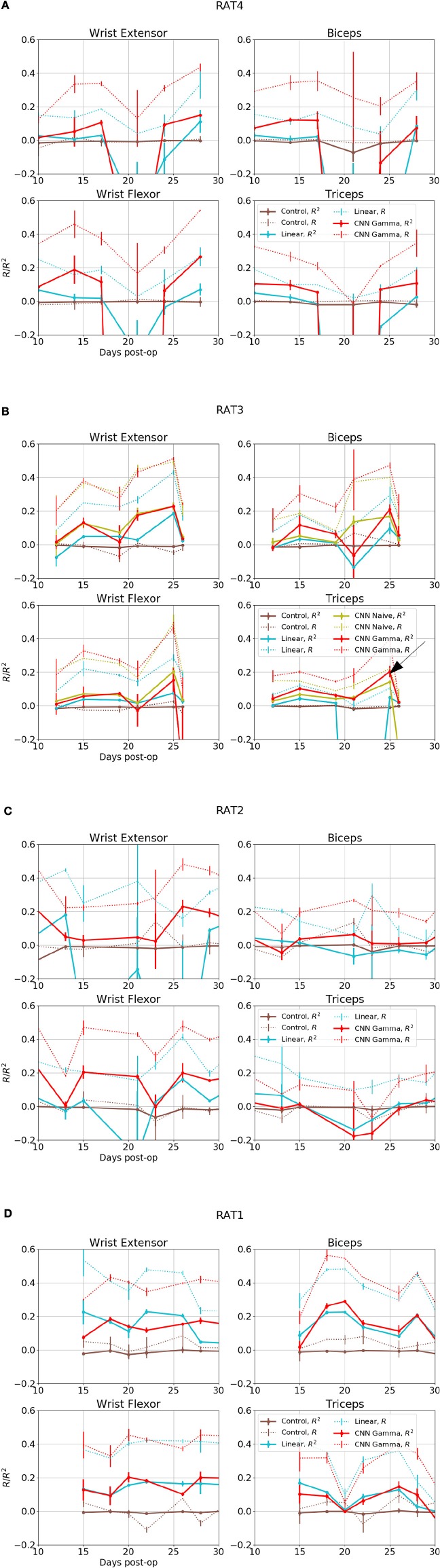
Overall performance for four EMG signals from 4 subjects over days. X axis is the days after electrode implant. Solid lines- *R*^2^, Coefficient of Determination, Dashed Lines- *R*, Correlation Coefficients. Red: Performance of Gamma Function Networks, Cyan, Control: Linear Decoder; Brown, Control: Gamma Function Network trained on shuffled inputs. **(B)** Green lines shows the performance of unrestricted CNN; black arrow in indicates the data set for the plots shown in the next figure.

Two-fold validation were a computationally efficient way to detect for variations caused by trial selections. Because multiple training attempts are required for each test set, it would have been costly to resample trials to generate robust statistics. the cross validation revealed large difference in performance between the two test set in some cases (Error bars in Figure 6).This identical procedure were also applied to controls described next for consistency.

### Control

**Figure 7:**
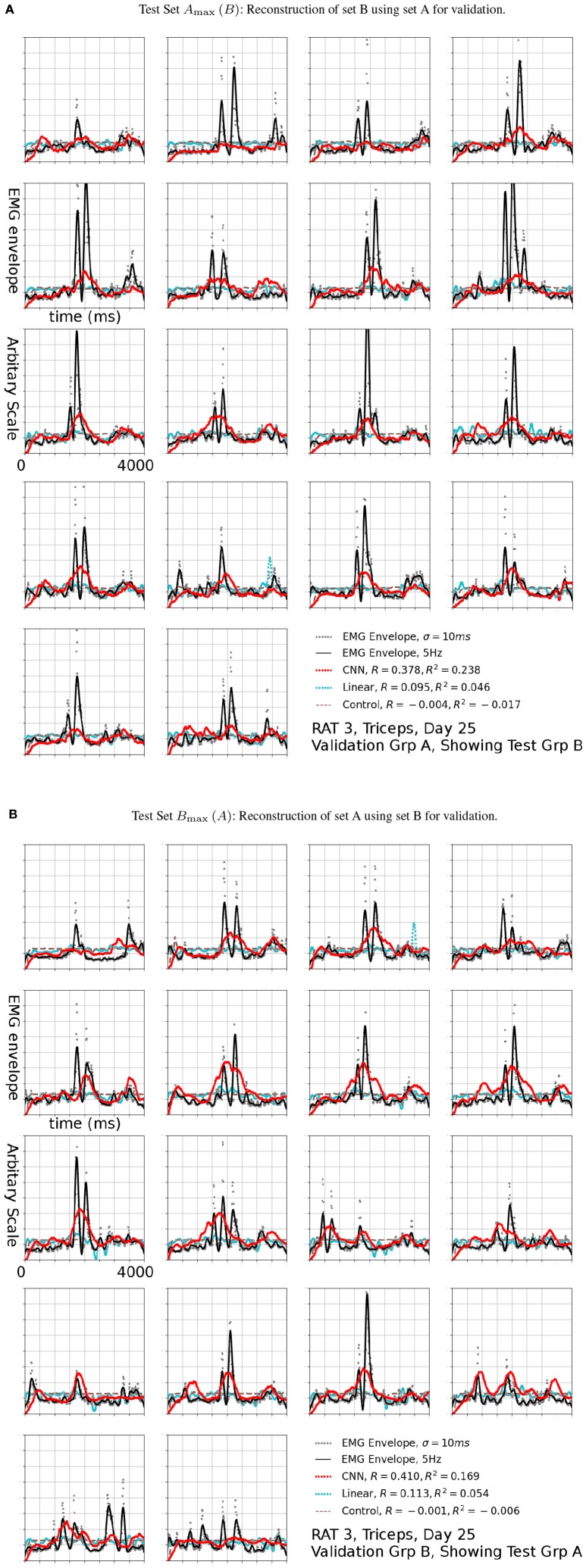
Reconstruction (Red, CNN; Cyan: Linear; Brown Dashed: Scrambled CNN Control) of low-pass filtered EMG signals (Black lines) from neural signals. Original EMG envelopes without low-pass filtering were drawn in gray dots for reference. This signal was marked by a black arrow in 6b. Scrambled Control (Brown): Training was carried out on scrambled singals, producing predictions that are essentially flat after the first transient. Horizontal axis: Time bins of 10 ms, each trial is 4 s long. This duration is identical for Figures 7, 9.

To confirm that the optimizer drew inference from neural data rather than fitting a model with slow transients, time permutation was used as a control. Time points of neural envelopes were randomly switched with another from the same or a different trial. This shuffle occurred as the last step of pre-processing, and did not reduce signal envelopes to DC. Sixteen control networks was trained on the scrambled input signal and produced reconstruction on intact test sets. The results of scrambled controls are shown in gray in Figures 6, 7, **9**.

To examine if neural networks captured complex relations that cannot be represented with simpler models, a linear decoder was included for comparison. For consistency, the linear decoder was implemented as a neural network of one layer and no rectification. Linear decoders received identical pre-processed inputs as the CNNs with one exception: Neural Evenlopes for linear decoders were low-pass filtered to the same frequency as the EMG signals (Ususally 5Hz). They would otherwise be very noisy. The results of linear decoders are shown in blue in Figures 6, 7, **9**. Given identical inputs, the differences between performances of linear and CNN decoders can be attributed to models with and without memory.

## 3. Results

Figure 6 shows overall performance for all subjects, sessions and EMG channels. The performance in reconstructing different EMG signals appeared to be correlated across days, suggesting that day-to-day electrode drift has a large impact on data quality. Because all EMG signals collected on the same day as a group were predicted well or otherwise, it suggest a common set of neural signals was required for the prediction and it was either present or completely absent.

The CNN visibly out-performed baseline linear decoders for rats 3 and 4 Figures 7A,B. Notable exceptions were sessions where some signals could not be reconstructed using any method (Rat 4 day 10 and 21, Rat 3 day 12 and 21). In those cases both methods had performance similar to or below the level of random controls or DC signals. Remarkably, for a number of sessions and EMG signals, the CNN model was able to reconstruct the EMG with *R*^2^over or close to 0.2, while the linear decoder could not reconstruct them at all (*R*^2^ below 0.05).

This is also observed in Rat 2, especially for Wrist Flexor. The CNN model also outperformed the linear decoder on Wrist Extensor and Biceps of this animal, except for Day 13 and 34 at the start and the end of the experiment. the CNN performed below both linear decoder and control for Triceps of this animal. However the linear decoder also had negative *R*^2^ for most sessions in this data set, suggesting an experimental issue. Performance of Linear and CNN models were comparable for Rat 1. In summary, CNN models did at least as well as the Linear Decoder except for Tricep of Rat 2, where both method had difficulties.

The *R*^2^ values appeared to be modest, but the variance errors were not distributed uniformly in time, i.e., a large amount of RMS error appeared to arose from a relatively small number of erroneously reconstructed time bins. This was demonstrated in Figure 7, which presents the test sets *A*_max_ (*B*) (the First 18 plots) and *B*_max_ (*A*) (the remaining half) for Triceps, Day 25, rat 3 (marked by the black arrow in Figure 6B). The CNN produced reconstructions that were qualitatively different from those generated by linear decoders as shown in cyan in Figure 7, the linear decoder appears to have only captured the rising edges of EMG bursts. However, the correlation was 0.378 and 0.410 for the first and second half, respectively.

**Figure 8:**
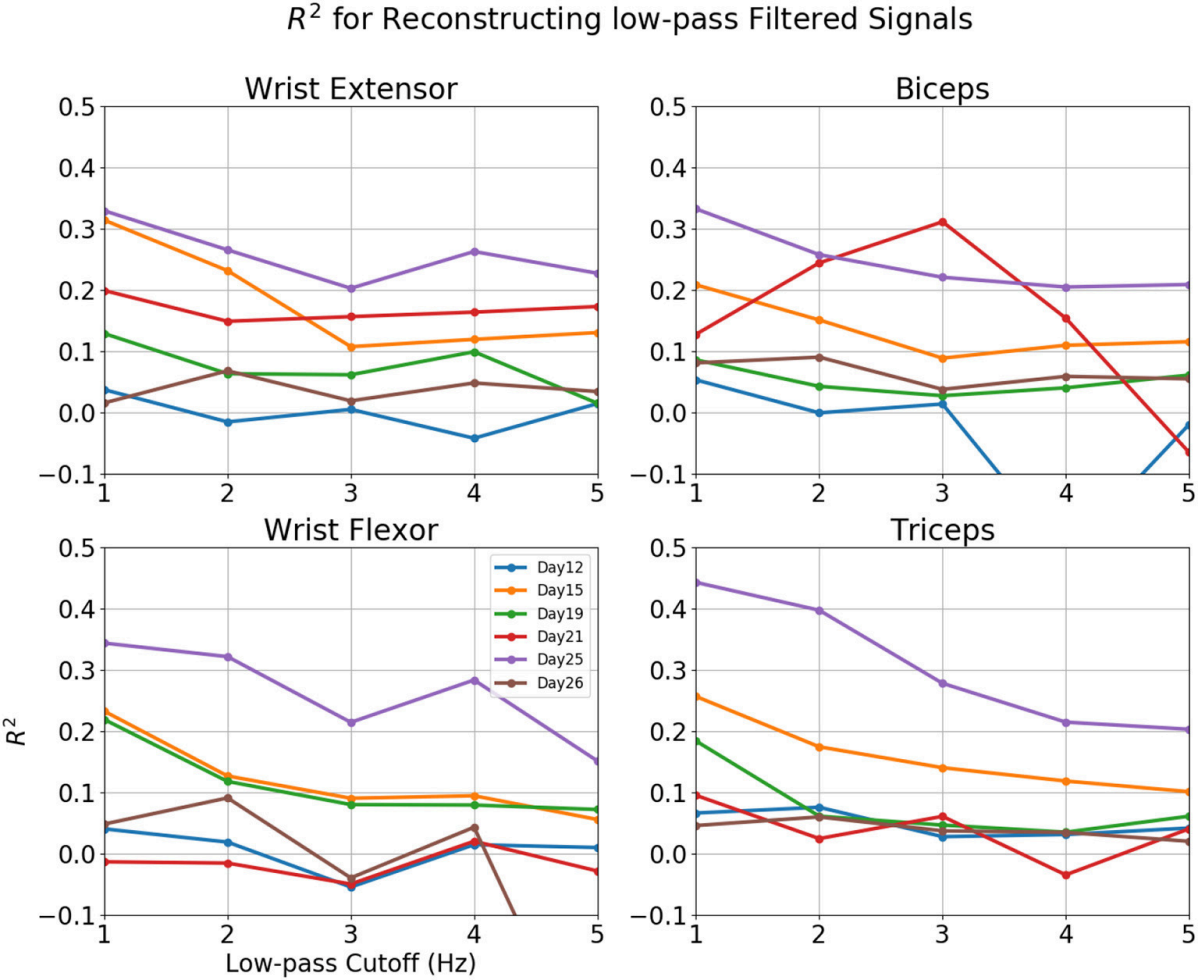
*R*^2^-value for reconstruction of low pass filtered EMG for various sessions of rat 3. Horizontal axis: Cut-off frequency for EMG, 0–5 Hz. Vertical Axis, *R*^2^. Each line represented a different session.

**Figure 9:**
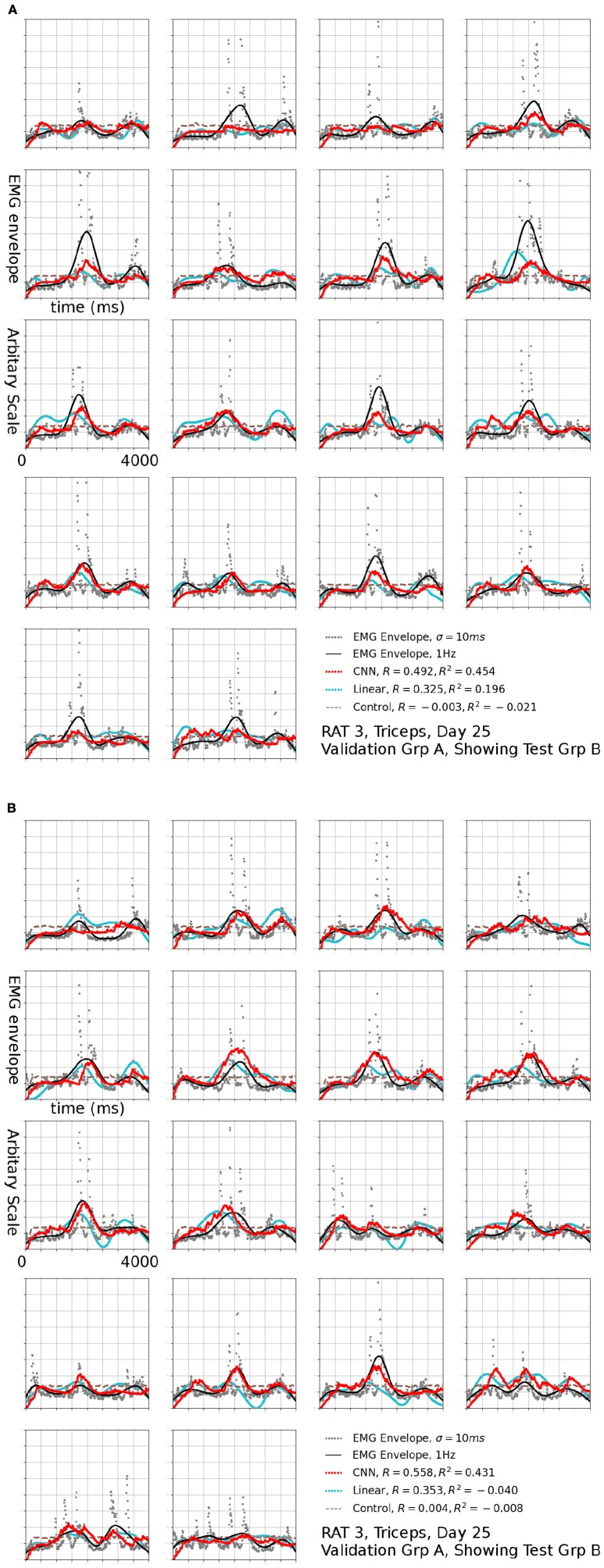
Reconstruction of set **(B)** using set **(A)** as validation, the EMG signal is the same one shown in Figure 7a, filtered to 1 Hz. The input to linear control were also filtered to 1 Hz.

This concentration of error was caused by the very uneven nature of the data. The model must accurately reconstruct the timing of EMG bursts. Otherwise, RMS errors quickly became very large whenever the network failed to reconstruct a burst, or if it predicted a burst at an incorrect time point. The standard *R*^2^ may not be the best measure of error in this kind of data because it considers only vertical distances and not horizontal ones (time delays). The results from 1Hz Figures 8, 9 supportted this. Perhaps some variation of minimum editing distance (Victor and Purpura, 1996; Schreiber et al., 2003) can provide a better measure in the future.

At a glance, the EMG profiles in each trial appears to be similar. This was caused by the alignment of trials (see section Materials and Methods). Stereotyped profiles raised concerns of whether the reconstructions were made using neural signals. This was addressed by the a control network, whose output is shown as a dashed brown line in Figures 7, 9. Because an initial segment of each trial did not factor into error calculations, and that segment was longer than the transient of the network, it was mathematically impossible to generate fits by transients.

When combined, the result shows that while CNN performed well only on some sessions, it would have been impossible to reach these performances by chance, considering the visible difference between the performances of CNN, and linear and random controls in these cases. The discussion section will speculate on the prediction performances achieved.

Reconstructions of some sessions were improved when the cutoff frequency of the low-pass filter applied to the EMG envelope was reduced. This effect is dramatic and *R*^2^ in some cases exceeded 0.4 at 1 Hz (Figure 8). A probable cause is that a low pass filter convert bursts into smoothed rates that made temporal errors less detrimental. This was also seen in Figure 9, where *R*^2^ = 0.454 for Set B and 0.431 for Set A. The observation that the model performed better at lower cut-off may have additional implications for interpreting the reconstruction, which will be discussed in detail in the next section.

**Figure 10:**
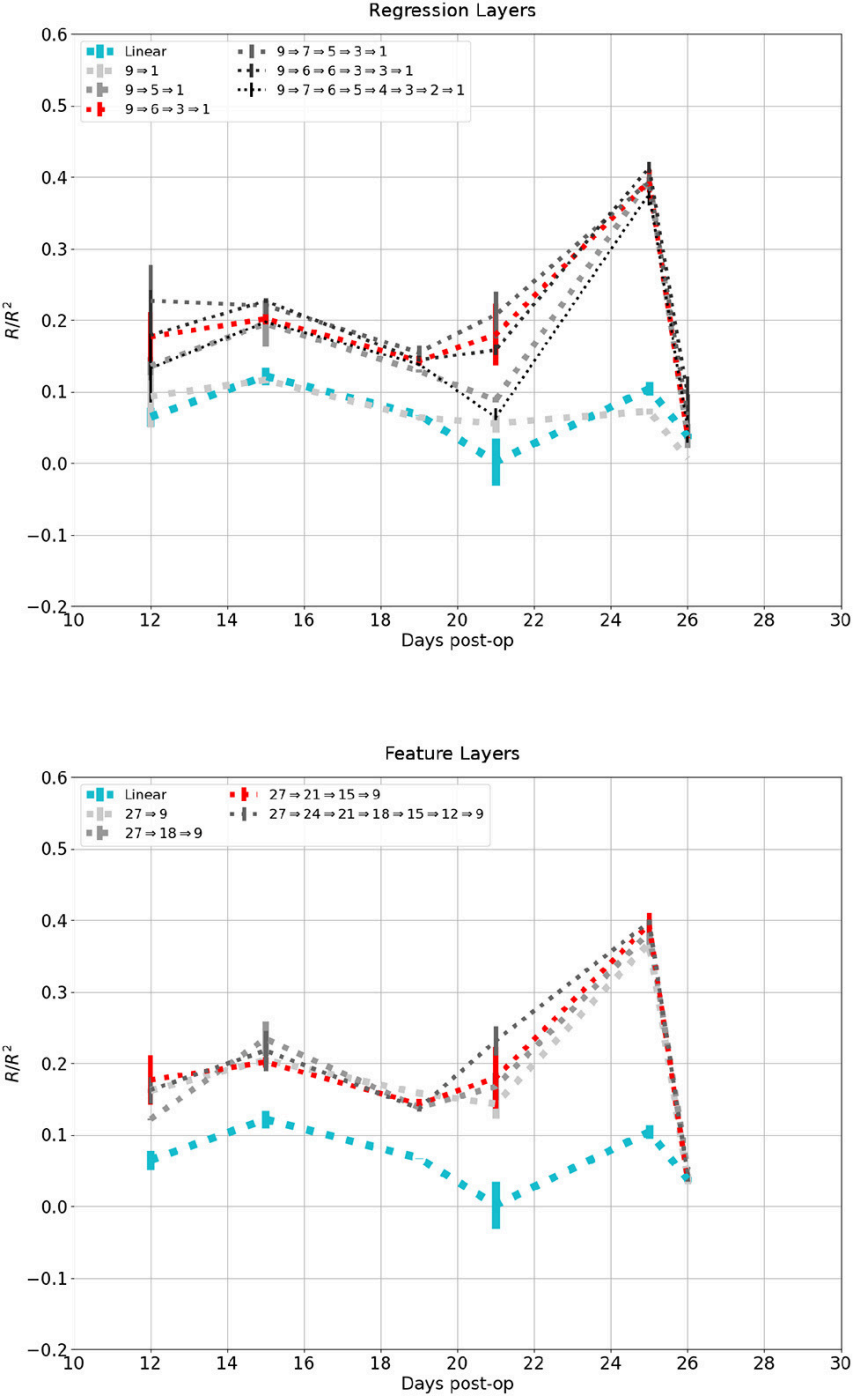
Reconstruction of the same signal in Figure 6B using increasing number of network layers. Darker and thinner lines corresponds to deeper networks. Red and Cyan line correspond to the network and linear control shown in previous figures. **Top**: Fixed feature layers, **Bottom**: Fixed Regression layers.

Figure 10 shows the affect network depth had on performance on the dataset in Figure 7. The top plot showed change in performance when the number of regression layers increased while session-dependent feature layers were kept constant. A network with a single layer had a performance similar to the linear control, while one with two layers performed similarly to one with three layers, except for the session at 21 days. additional layers lead to diminishing return and eventually degradation in performance. The lower plot showed change in performance when the number of feature layers were increased while regression layers were kept constant. Networks with 1, 2, or 3 feature layers all performed similarly, while a network with 6 feature layers performed marginally better.

Gamma function networks were still able to train when sampling rate was set to 1kHz, producing a reconstruction with *R*^2^ = 0.202, *R* = 0.334 for test set B of the dataset in Figure 7 in four attempts. while a naive CNN without closed form did not converge after 16 attempts due to the increased number of parameters. Therefore, a closed form improved the network's ability to converge and computational performance. At sampling rate of 100 Hz, both networks converged and produced similar reconstructions (Red vs. Green Line in Figure 6B).

## 4. Discussions

### Gamma function networks

The CNNs in this study were organized unconventionally as an adaptation to neural data. Unlike images, the time dimension of neural (and EMG) data is much greater than its feature dimension, and there appeared to be little spatial structure within the feature dimension. This is consistent with the data presentation in Schirrmeister et al.'s (2017) paper (Schirrmeister et al., 2017). Conventionally, the CNNs employ small filters with limited span to discover features embedded in the data in a hierarchy (Krizhevsky et al., 2012). While this method worked, training was not straightforward. It required the data to be sampled at a low frequency and kernel length to be limited to avoid overwhelming complexity.

Complexity of neural network models must be match the amount of data available. Because the ANNs approximate the solution by cascade of activation functions (Hecht-Nielsen, 1989), optimizers can draw connections in the training data unrelated to true mechanisms and are not predictive of the test data. The more complex the network, the more training data is required to prevent this. The best practice established is to let the optimizer select network parameters such as the shape of filters, however when training data is limited, this would result in parameters that can quickly over-fit the training set.

The Gamma functions improved performance by reducing the number of parameters to be estimated. This reduced over-fitting when working with limited data. Unlike L1 or L2 regularizations, an analytic function carries the implicit assumption that layer responses are continuous. The reduced complexity allowed a network comprised of analytic functions to train without constraining kernel length using prior knowledge of probable system response times. Because analytical functions retain the same number of parameters regardless of the sampling rate, a CNN of analytical functions could work at higher sampling frequencies, while an unrestricted CNN cannot.

The gamma function was initially chosen because it resembled the shape of excitatory post-synaptic potentials (EPSP), thus presented a resemblance for construction of muscular action potentials and end-plate potentials. However, this approached most likely worked for a different reason in practice because diagnostics showed that the output signals were an order of magnitude longer than neuro-muscular end-plate potentials in the rat Figure 4. It is possible that any one sided unimodal functions would perform equally well.

### Relations to past work

The result showed that Convolutional ANN are capable of predicting EMG activities during skilled movements. Unlike in the previous study (Guo et al., 2014), this paper was concerned largely with free movements where the limb position was unknown.

Due to the presence of the spinal reflex pathways that include the peripheral feedback, neural signal alone is insufficient to predict EMG signals in unrestricted movements. The spinal cord expresses a stretch reflex, where passively stretching muscle causes them to contract to resist the stretch (Ghez and Shinoda, 1978). This reflex is caused by muscle spindles activating alpha motor neurons and can occur in the absence of voluntary command. The stretch reflex is postulated to stabilize postures and movements against external perturbations (Feldman, 1986; Levin and Feldman, 1994).

Because of this, movements are jointly determined by descending signals, mechanical properties of the limbs, and the local feedback circuits in the spinal cord. Identical descending signals may result in different EMGs depending on external factors. However, it is possible to treat this entire system, including downstream mechanics and feedback, as a non-linear FIR filter and perform system identification in the context of stereotyped, skilled movements. For instance, a model could detect a shift in neural signal encoding intentions, and predict an EMG bursts of certain size and duration based on transient system response to similar shifts without knowledge of limb positions. These predictions would be correct as long as the animal performed the same kind of movement.

Speculatively, the presented Convolutional Neural Network (CNN) contained more than a model of neuromuscular activation for two reasons. Firstly, the optimizer produced Gamma functions which can persists for over 50ms per layer, leading to model responses that can last over 100 ms Figure 4. Considering the neural propagations times to the muscles implanted with EMG electrodes in the rat (<10 ms), this response time is much longer than expected neuromuscular activation delay and duration.

Secondly, point to point linear decoders (without temporal memory) were frequently ineffective at similar cutoff frequencies (Red vs. Cyan in Figures 6, 7, 9). This suggests no simple instantaneous mapping exist between the neural signal and EMG.

Together, these factors suggest that the ANN made implicit predictions of movements, possibly fast transient mechanics of limbs in response to changes in command signals. At this time, it is not yet clear which aspects of the movements were captured by the networks because ANN models are black-boxes for parametric analysis. Therefore, the next logical step for this project will be to apply CNN to data generated by an analytical biomechanic model (Hogan, 1985; Hogan and Sternad, 2012) that can be manipulated to test the limits of the CNN's capabilities.

### Future directions

Recurrent architecture is another possible direction for improvement. This can be pursued in two ways. The first is autoregressive models. The difference between predicted and measured EMG signals may contain information about system dynamics, such as limb velocities, which may be used to make better predictions. Autoregressive models can take advantage of this difference, however care must be taken to account for self-correlation in EMG signals in time. Recent advances in Sequential Convolution Architectures Such as ByteNet (Kalchbrenner et al., 2016) SliceNet (Kaiser et al., 2017) and PoseNet (Chen and Wu, 2017) makes this a promising avenue of future investigation.

Alternatively, one could adopt fully recurrent network architectures like Long Short Term Memory (LSTM) (Gers Jj et al., 1999) or Deep Autoregressive Network (DARN) (Gregor et al., 2014) These may work better than convolutional ones if the context predicts movements better than timing. Recurrent Neural Network such as LSTM have persistent states, which are only replaced under some specific input contexts (specific features which cause write gate to open). These networks do not require predicting (neural) features to be at a fixed distance to the predicted (EMG) features. While powerful, these networks are also very difficult to train with limited data, because an EMG burst could potentially be explained by any neural features occurring beforehand.

Beside testing alternative ANN architectures, it will be important to examine whether orthogonal basis function expansion (Marmarelis, 1993; Chan et al., 2009) can produce a better solution. Basis function expansions can be thought as a two layer equivalent network - the first layer convolves inputs with a series of fixed orthogonal waveforms, and the results of these convolutions and their products enter a densely connected output layer with logistic activation. Speculatively, multiple layers of Gamma functions can represent a similar set of non-linearities as Laguerre basis functions, but a study is still required to verify this.

Other than sessions manually rejected due to excessive artifact contamination, there did not appear to be parameters (date or length) that effectively predicted performance of a session. Session performances were also largely unaffected by depth of the network or number of features extracted by session dependent layers Figure 10. Attempts were also made to train the network to select appropriate frequency bands by modulating the Gaussian Envelope (see reprocessing) with sinusoidal signal from 1 to 500 Hz. This produced 20 signals per neural channel and the resulting 540 signals were fed into either spatial convolutions or densely connected layers. Both approaches slightly decreased the performance, possibly because they drastically increased the complexity of the feature layers, and each session did not contain a large amount of data. Results of these explorations suggested that causes for performance variability were experimental.

While some evidence suggest that this may have been caused by micro-motion of the electrode contact with respect to neural tissue carring the relevant information, additional data will be necessary to determine the exact nature of these variability. Long episodes of continuous recording may also be effective way to increase data availability, regardless of the behavior that the animals are engaged in, as long as they are active. Data dominated by long periods of silent neural signals are very unbalanced however, and will require additional preprocessing.

An advantage of CNN models is that they rely on matrix operations already implemented in existing graphics acceleration hardware. In this project, One training step for one EMG signal and one animal took 55–62 ms with sampling period of 10 ms and approximately 1,500 ms with the sampling period of 1 ms. However, predictions took much less time than a single training step because only the forward pass was needed. For all the practical purposes predictions were instantaneous. An embedded system with on-board processing power equivalent of a modest laptop graphics processor is in theory sufficient to run the predictions in real time. While the initial training is slow, it can be carried out over days or weeks on an embedded or implantable system, without transferring a large amount of data. This will only require external supervision to switch the model from training to predictive mode.

## 5. Conclusions

CNNs were able to reconstruct EMG signals from neural signals recorded from the CST tract of freely moving rats for selected sessions, well above the level of linear decoders. Additional evidences suggest that this was probably accomplished by an implicit prediction of movement dynamics. These result shows variations of the CNNs defined with analytical functions and using session-to-session transfers may present powerful options for decoding neural signals, as long as testable controls can be formulated to account for alternative interpretations. Analytical mechanistic models as well as additional data will help in better interpreting the results. CNN models can operate on existing graphics processing hardware, making it possible to develop efficient standardized self-contained implantable systems to house these models for real-time applications.

## Ethics statement

All procedures were approved by the Institutional Animal Care and Use Committee (IACUC), Rutgers University, Newark, NJ.

## Author contributions

YG: Deep learning algorithm and manuscript drafting; SG: Data collection and proofreading; MS: Project supervision.

### Conflict of interest statement

YG is the founder and the sole owner of Hybrid Intelligence Laboratories LLC. The remaining authors declare that the research was conducted in the absence of any commercial or financial relationships that could be construed as a potential conflict of interest.
